# Mental and behavioral disorder mortality in Poland, 2000–2023: a national age-standardized Joinpoint regression study

**DOI:** 10.3389/fpubh.2025.1731183

**Published:** 2026-01-12

**Authors:** Małgorzata Pikala

**Affiliations:** Department of Epidemiology and Biostatistics, the Chair of Social and Preventive Medicine of the Medical University of Lodz, Lodz, Poland

**Keywords:** alcohol, causes of death, dementia, mental and behavioral disorders, mortality trends, Poland

## Abstract

**Introduction:**

Many studies on mental health show that mental and behavioral disorders (MBD) are becoming a growing global problem, including in Poland. The aim of the study is to assess mortality trends due to MBD in Poland in the period 2000–2023, by gender and by the most important causes in this group of deaths.

**Methods:**

The study material was a database including 63,580 death certificates of all Polish inhabitants who died due to MBD in the period 2000–2023. The authors calculated standardized death rates (SDR), annual percentage change (APC) and the average annual percentage change (AAPC).

**Results:**

The number of deaths due to MBD in Poland increased from 1,541 in 2000 to 5,018 in 2023. The standardized death rate (SDR) increased from 4.70 to 13.42 per 100,000 population (AAPC = 5.2%, *p* < 0.05). SDR increased more rapidly in women, i.e., from 1.50 to 8.09 (AAPC = 7.5%, *p* < 0.05) than in men, i.e., from 8.40 to 19.06 (AAPC = 4.3%, *p* < 0.05). Over 80% of all deaths in this group were caused by MBD due to alcohol use. SDR trends for these causes began to increase statistically significantly from 2013 at an annual rate of 8.1% (*p* < 0.05), reaching 6.9 in 2023. In the male group, APC in 2013–2023 was 8.3% (*p* < 0.05), and SDR in 2023 was 12.9. In the female group, the APC in 2014–2020 was 13.1% (*p* < 0.05), and SDR in 2023 was 1.7. A statistically significant increase in SDR due to dementia was also observed from 2011. APC between 2011 and 2023 was 26.4% (23.6% in the male group and 27.9% in the female group). In 2023, SDR was 5.04 (4.34 and 5.25, respectively).

**Conclusion:**

The increase in mortality due to MBD related to excessive alcohol consumption and population aging is a growing public health challenge that requires systemic intervention.

## Introduction

Many studies on mental health show that mental and behavioral disorders (MBD) are a serious and growing global problem. According to WHO estimates, approximately one in eight people worldwide is affected by a mental disorder (1). In 2016, 283 million people suffered from alcohol abuse-related disorders (2), 36 million people had drug abuse-related disorders (3), 55 million people had dementia (4), and 24 million people had schizophrenia (5).

MBD occur slightly more frequently in high-income countries (15.1%) than in low-income countries (11.6%) (1). In Europe, the rate is 14.2%. Differences in prevalence rates across regions and income groups are mainly due to demographic reasons. The burden of MBD spans the entire life cycle: from early age, where conditions such as developmental disorders and childhood behavioral disorders are the greatest contributors to the burden. They occur until adulthood and old age, where depressive and anxiety disorders as well as dementia predominate. In consequence, MBD are the leading cause of years lived with disability (YLDs), accounting for one in every six YLDs globally (1).

The most serious consequence of MBD is death. Worldwide, mortality rates in people with MBD are disproportionately higher compared to the general population (6). People with severe MBD die on average 10 to 20 years earlier than others (7).

MBD not only directly affect sufferers and their families, but also entail enormous economic costs for society. In addition to direct costs of treatment, MBD are associated with a range of indirect costs related to reduced economic productivity, higher unemployment rates, and other economic effects. These costs to society often far exceed the costs of healthcare. Researchers of the World Economic Forum have calculated that a broadly defined set of MBD cost the global economy approximately $2.5 trillion in 2010. Of this number, $1.7 trillion was associated with a loss of economic productivity whereas $0.8 trillion was connected with direct care costs (8).

Most countries around the world are taking measures to improve the mental health of their citizens. In Poland, the National Mental Health Protection Program for 2023–2030, established under the Regulation of the Council of Ministers of November 15, 2023, is currently in force (9). The main objectives of this program include providing people with MBD, including those with addictions and experiencing mental crises, support and care appropriate to their needs, taking measures to prevent stigmatization and discrimination against people with MBD, developing mental health centers, and introducing a new model of mental health care for children and adolescents.

Implementation of measures aimed to improve life of mentally disturbed people should be preceded by an accurate epidemiological diagnosis so that interventions are tailored to trends and needs in this area.

The aim of this study was to assess trends in mortality due to MBD in Poland in the years 2000–2023, by gender and the most important causes in this group of deaths.

## Materials and methods

The study material was a database including 63,580 death certificates of all Polish inhabitants who died due to MBD in the period 2000–2023 (according to the International Statistical Classification of Diseases and Health Related Problems—Tenth Revision—ICD-10, coded as F00-F99). The analysis also identified three most important causes of death, i.e., dementia (F01-F03), MBD due to alcohol use (F10) and schizophrenia (F20). The data were provided by Statistics Poland.

The procedure of coding causes of death in Poland is performed in a similar way to the one carried out in the majority of countries in the world, by basing on the so called primary cause of death, or the disease which triggered a pathological process, leading to death.

The authors calculated standardized death rates (SDR). The standardization procedure was performed with the use of direct method, in compliance with the European Standard Population, updated in 2013 (10).

The analysis of time trends has been carried out with joinpoint models and Joinpoint Regression Program, a statistical software package developed by the US National Cancer Institute for the Surveillance, Epidemiology and End Results Program (11).

The joinpoint regression model is an advanced version of linear regression y = bx + a, where: b is the slope coefficient, a is the y-intercept, y is a measure evaluated in the study and x is a calendar year. Time trends were determined with the use of segments joining in joinpoints where trend values significantly changed (*p* < 0.05). In order to determine whether the changes were statistically significant, the Monte Carlo Permutation method was applied.

Besides, the authors also calculated the Annual Percentage Change (APC) for each segment of broken lines and Average Annual Percentage Change (AAPC) for the whole study period with corresponding 95% confidence intervals (CI).

The Annual Percent Change is used to characterize trends in death rates over time and it was calculated according to the following formula:


$$\mathrm{APC}=100*(\exp^{b}–1)$$

Where: b—the slope coefficient.

The Average Annual Percent Change (AAPC) is a summary measure of the trend over a pre-specified fixed interval. It allows us to use a single number to describe average APCs over a period of multiple years. It is valid even if the joinpoint model shows that there were changes in trends during those years. It is computed as a weighted average of the APCs from the joinpoint model, with the weights equal to the length of the APC interval (12).


$$\text{AAPC}=\{\exp(\frac{\sum w_{i}b_{i}}{\sum w_{i}})–1\}\times 100$$

Where: b_i—_the slope coefficient for each segment in the desired range of years, w_i_—the length of each segment in the range of years.

Statistical analyses were performed using the Statistica (data analysis software system), version 13.5 (TIBCO Software Inc.) (13).

## Results

**Figure 1:**
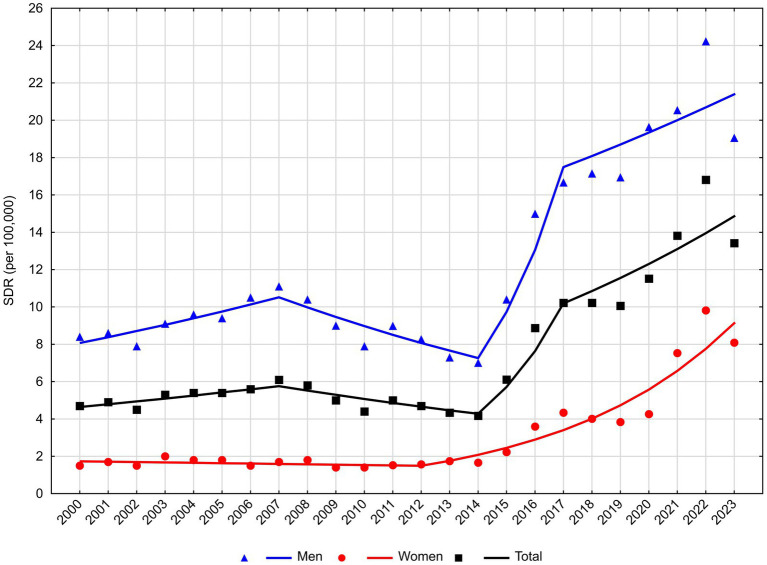
Trends in standardized death rates for mental and behavioral disorders in Poland, 2000**–**2023.

Between 2000 and 2023, 63,580 people died in Poland due to MBD. The annual number of deaths due to these causes increased from 1,541 in 2000 to 5,018 in 2023 (Table 1). Standardized death rates (SDR) per 100,000 population increased from 4.70 to 13.42, respectively. Between 2000 and 2007, the Annual Percentage Change (APC) increased insignificantly, while SDR rates appeared to decrease insignificantly between 2007 and 2014 (Table 2; Figure 1). From 2014, there was a very rapid, statistically significant increase in SDR at an annual rate of 33.6%. In 2017, the annual rate of increase slowed, but remained high, i.e., 6.5% (*p* < 0.05). The Average Annual Percentage Change (AAPC) throughout the entire observation period was 5.2% (*p* < 0.05).

**Table 1:** Number of deaths and standardized death rates (SDR) due to mental and behavioral disorders in Poland, 2000–2023.

Year	Total		Men		Women	
	Number of deaths	SDR (per 100,000)	Number of deaths	SDR (per 100,000)	Number of deaths	SDR (per 100,000)
2000	1,541	4.70	1,295	8.40	246	1.50
2001	1,594	4.90	1,332	8.60	262	1.70
2002	1,485	4.50	1,231	7.90	254	1.50
2003	1,705	5.30	1,391	9.10	314	2.00
2004	1,811	5.40	1,520	9.60	291	1.80
2005	1,811	5.40	1,516	9.40	295	1.80
2006	1,967	5.60	1,702	10.50	265	1.50
2007	2,156	6.10	1,831	11.10	325	1.70
2008	2,077	5.80	1,745	10.40	332	1.80
2009	1,859	5.00	1,576	9.00	283	1.40
2010	1,785	4.40	1,489	7.90	296	1.40
2011	1,859	5.00	1,557	8.99	302	1.52
2012	1,708	4.70	1,407	8.27	301	1.57
2013	1,546	4.34	1,218	7.30	328	1.74
2014	1,495	4.17	1,181	7.01	314	1.66
2015	2,268	6.11	1,828	10.40	440	2.22
2016	3,204	8.87	2,489	14.99	715	3.59
2017	3,723	10.22	2,851	16.67	872	4.34
2018	3,762	10.22	2,932	17.15	830	4.01
2019	3,807	10.06	3,002	16.95	805	3.84
2020	4,387	11.52	3,500	19.64	887	4.26
2021	5,050	13.82	3,439	20.54	1,611	7.53
2022	5,962	16.81	3,884	24.23	2,078	9.82
2023	5,018	13.42	3,210	19.06	1,808	8.09
Total	63,580		49,126		14,454	

**Table 2:** Time trends of SDR due to mental and behavioral disorders in Poland, 2000–2023—joinpoint regression analysis.

Sex	Number of joinpoints	Years	APC (95% CI)		AAPC (95% CI)	
Mental and behavioral disorders (F00-F99)						
Total	3	2000**–**2007	3.1	(−0.4; 6.8)	5.2*	(1.4; 9.1)
		2007**–**2014	−4.2	(−8.3; 0.2)		
		2014**–**2017	33.6*	(2.6; 73.9)		
		2017**–**2023	6.5*	(1.8; 11.3)		
Men	3	2000**–**2007	3.9*	(0.4; 7.4)	4.3*	(0.8; 8.0)
		2007**–**2014	−5.2*	(−9.1; −1.1)		
		2014**–**2017	34.0*	(4.4; 72.2)		
		2017**–**2023	3.4	(−0.9; 7.9)		
Women	1	2000**–**2012	−1.2	(−4.0; 1.6)	7.5*	(5.4; 9.7)
		2012**–**2023	17.9*	(14.1; 21.8)		
Dementia (F01-F03)						
Total	1	2000**–**2011	−5.3	(−11.6; 1.3)	10.1*	(5.5; 14.8)
		2011**–**2023	26.4*	(19.1; 34.2)		
Men	1	2000**–**2011	−5.7	(−12.8; 2.1)	8.6*	(3.4; 14.0)
		2011**–**2023	23.6*	(15.3; 32.4)		
Women	1	2000**–**2011	−5.1	(−12.1; 2.4)	10.9*	(5.8; 16.2)
		2011**–**2023	27.9*	(19.7; 36.7)		
Mental and behavioral disorders due to alcohol use (F10)						
Total	1	2000**–**2013	1.2	(−1.5; 4.1)	4.2*	(1.9; 6.5)
		2013**–**2023	8.1*	(3.7; 12.6)		
Men	1	2000**–**2013	0.6	(−2.1; 3.5)	3.9*	(1.6; 6.2)
		2013**–**2023	8.3*	(3.9; 12.8)		
Women	2	2000**–**2014	4.8*	(2.9; 6.8)	4.3*	(0.9; 7.9)
		2014**–**2020	13.1*	(3.6; 23.4)		
		2020**–**2023	−13.1	(−28.5; 5.8)		
Schizophrenia (F20)						
Total	1	2000**–**2012	−19.7*	(−26.4; −12.4)	2.5	(−3.6; 9.1)
		2012**–**2023	33.9*	(21.2; 47.8)		
Men	1	2000**–**2013	−18.8*	(−26.6; −10.2)	4.3	(−3.8; 13.1)
		2013**–**2023	44.4*	(24.4; 67.7)		
Women	1	2000**–**2011	−27.1*	(−34.4; −19.0)	1.4	(−5.1; 8.2)
		2011**–**2023	37.2*	(25.0; 50.4)		

*p < 0.05.

Mortality due to MBD is significantly higher in men than in women. The number of male deaths between 2000 and 2023 was 49,126 (Table 1). Between 2000 and 2023, the mortality rate increased from 1,295 deaths to 3,210 deaths. SDR increased from 8.40 to 19.06 per 100,000 population, respectively. The rate and direction of change in SDR changed three times (Table 2; Figure 1). Between 2000 and 2007, APC was 3.9% (*p* < 0.05), while between 2007 and 2014, a decline of −5.2% per year (*p* < 0.05) was observed. Between 2014 and 2017, there was a very rapid increase in SDR at an annual rate of 34.0% (*p* < 0.05), and after 2017, changes in SDR in the male group were not statistically significant. The Average Annual Percentage Change over the entire observation period was 4.3% (*p* < 0.05).

The number of female deaths between 2000 and 2023 was 14,454. It increased from 246 to 1,808 in this period (Table 1). Per 100,000 women, SDR increased from 1.50 to 8.09, respectively. Between 2000 and 2012, changes in SDR were not statistically significant, but from 2012, there was a rapid, statistically significant increase at a rate of 17.9% per year (Table 2; Figure 1).

A more detailed analysis of the causes of death reveals that over 80% of them were contributed by MBD related to alcohol (Table 3). The second most common cause was dementia, which caused 14.6% of deaths. Schizophrenia caused 1.8% of deaths, while drug dependence and toxicomania caused 0.5%. The structure of causes of death differed significantly between the sexes. Deaths due to MBD due to alcohol abuse dominated in the male group (90.7%), while in the female group, the percentage of deaths due to these causes was 45.3%. Dementia was significantly more likely to cause deaths in women (44.1%) than in men (5.9%). The third most common cause, i.e., schizophrenia, also caused more deaths among women (4.0%) than men (1.1%).

**Table 3:** Major causes of mortality from mental and behavioral disorders in Poland, 2000–2023.

Causes of mortality	Total		Men		Women	
	Number of deaths	%	Number of deaths	%	Number of deaths	%
Mental and behavioral disorders due to alcohol use (F10)	51,114	80.4	44,570	90.7	6,544	45.3
Dementia (F01-F03)	9,285	14.6	2,907	5.9	6,378	44.1
Schizophrenia (F20)	1,125	1.8	547	1.1	578	4.0
Drug dependence, toxicomania (F11-F16, F18-F19)	334	0.5	235	0.5	99	0.7
Other causes (F04-F09, F17, F21-F99)	1,722	2.7	867	1.8	855	5.9
Total (F00-F99)	63,580	100.0	49,126	100.0	14,454	100.0

**Figure 2:**
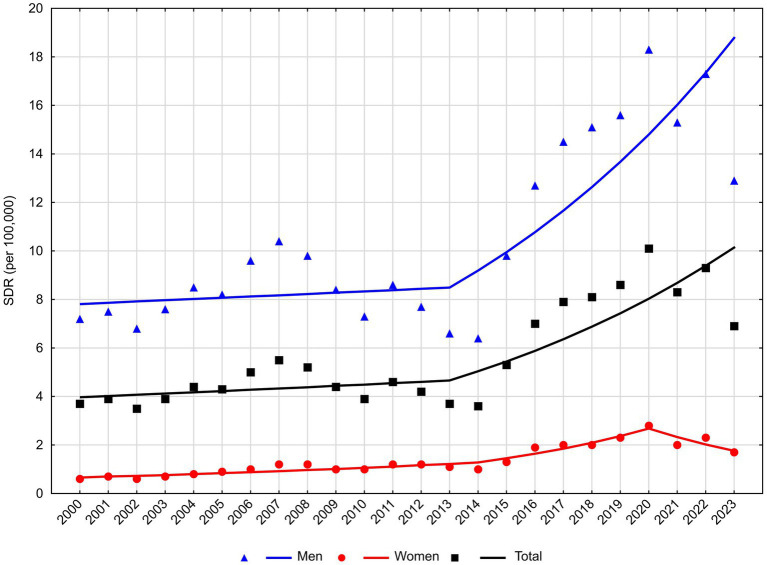
Trends in standardized death rates for mental and behavioral disorders due to alcohol use in Poland, 2000**–**2023.

An increase in the number of deaths due to MBD due to alcohol abuse occurred throughout the entire observation period (AAPC = 4.2%), with statistically significant changes beginning in 2013. APC for the years 2013–2023 was 8.1% (Table 2; Figure 2). In the male group, after statistically insignificant changes that were observed in 2000–2013, SDR began to increase at a rate of 8.3% (*p* < 0.05). In the female group, a statistically significant increase was observed between 2000 and 2020. The increase rate was 4.8% until 2014 and it accelerated to 13.1% after 2014. A statistically insignificant decline in SDR began in 2020.

**Figure 3:**
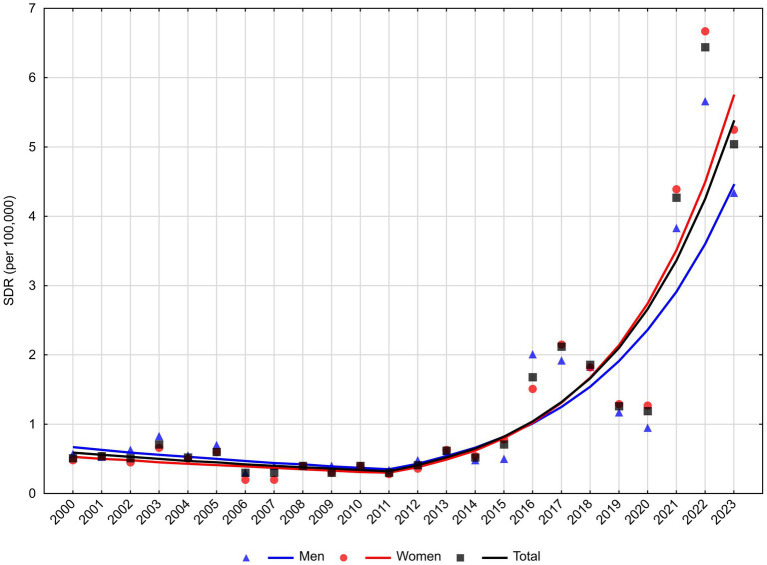
Trends in standardized death rates for dementia in Poland, 2000**–**2023.

Mortality rates due to the second most common cause, i.e., dementia, after a statistically insignificant decline in 2000–2011, began to increase rapidly at an annual rate of 26.4% (Table 2). This increase was observed in all genders, but was slightly faster in women (APC = 27.9%, *p* < 0.05) than in men (APC = 23.6%, *p* < 0.05; Figure 3).

**Figure 4:**
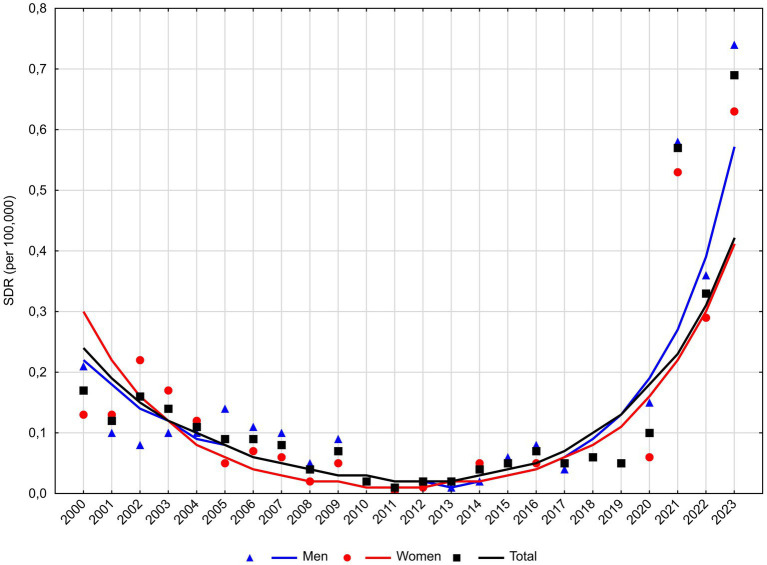
Trends in standardized death rates for schizophrenia in Poland, 2000–2023.

The third leading cause of death, i.e., schizophrenia, caused 1,125 deaths between 2000 and 2023, including 547 men and 578 women (Table 3). The SDR values for schizophrenia were relatively low, i.e., 0.17 in 2000 and 0.69 in 2023 (Figure 4). However, it is worth noting that from 2012 SDR due to schizophrenia was rapidly increasing (33.9% annually; *p* < 0.05; Table 2). This increase affected all sexes—it was slightly faster in the group of men (APC = 44.4%, *p* < 0.05) than in the group of women. (APC = 37.2%, *p* < 0.05).

The study confirmed the average age of people who died from MBD increased. In 2000, the average age at death was 53.8 years, and in 2023 it rose to 68.3 years (Table 4). The average age of death was higher in women (61.6 years in 2000 and 79.0 years in 2023) than in men (52.3 and 62.2 years, respectively).

**Table 4:** Mean and median age at death from mental and behavioral disorders in Poland in 2000 and 2023.

Sex	Mean ± SD		Median (Q1-Q3)	
	2000	2023	2000	2023
Mental and behavioral disorders (F00-F99)				
Total	53.8 ± 13.2	68.3 ± 16.8	51.0 (43.0–65.0)	68.0 (57.0–82.0)
Men	52.3 ± 11.8	62.2 ± 14.4	51.0 (44.0–61.0)	63.0 (52.0–71.0)
Women	61.6 ± 17.1	79.0 ± 15.3	61.0 (48.0–76.0)	84.0 (70.0–91.0)
Dementia (F01-F03)				
Total	78.0 ± 9.8	85.6 ± 8.3	77.0 (72.0–85.0)	86.0 (79.0–90.0)
Men	75.5 ± 10.7	81.8 ± 9.2	76.0 (71.0–83.0)	83.0 (75.0–89.0)
Women	79.5 ± 8.9	87.1 ± 7.4	78.0 (75.0–88.0)	88.0 (83.0–92.0)
Mental and behavioral disorders due to alcohol use (F10)				
Total	51.2 ± 10.5	58.6 ± 11.5	50.0 (44.0–59.0)	61.0 (51.0–67.0)
Men	51.2 ± 10.5	58.4 ± 11.5	50.0 (44.0–59.0)	60.0 (50.0–67.0)
Women	51.5 ± 10.1	59.7 ± 11.4	50.0 (45.0–58.0)	62.0 (51.0–68.0)
Schizophrenia (F20)				
Total	60.7 ± 11.5	67.2 ± 12.5	63.0 (54.0–69.0)	69.0 (61.0–75.0)
Men	58.3 ± 11.6	63.4 ± 13.2	61.5 (49.0–66.5)	66.0 (58.0–72.0)
Women	63.7 ± 10.8	70.5 ± 10.8	65.0 (60.0–70.0)	71.0 (63.0–77.0)

The increase in the average age at death between the first and last year of the study period 2000–2023 applied to all analyzed causes of death.

The highest average age at death was noted in people who died from dementia, i.e., 78.0 years in 2000 and 85.6 years in 2023. In the female group, the average age increased from 79.5 to 87.1 years, and in the male group from 75.5 to 81.8 years.

The lowest average age of death was noted among people who died from due to alcohol abuse. It was 51.2 years in 2000 and 58.6 years in 2023. These average values were similar in all gender groups.

The average age of people who died from schizophrenia in 2000 was 60.7 years and increased to 67.2 years in 2023. Men died from schizophrenia younger than women. The average age of death for men was 58.3 years in 2000 and 63.4 years in 2023. In the female group, these values were 63.7 and 70.5 years, respectively.

## Discussion

MBD—particularly severe mental disorders (SMD)—are associated with poorer health outcomes and increased mortality. SMD are defined as a group of conditions that include moderate to severe depression, bipolar disorder, and schizophrenia and other psychotic disorders. People with SMD die, on average, 10 to 20 years earlier than the general population (14). This premature mortality has been well documented in meta-analyses and systematic reviews (15–21).

The majority of deaths among people with MBD result from preventable physical diseases, particularly cardiovascular diseases (22, 23), respiratory diseases (24), infections (25), diabetes (16), and cancers (26).

The remaining deaths among people with SMD are due to unnatural causes, including suicide, homicide, and accidents (17, 27, 28).

MBD are a major public health problem in Poland. A study being a part of the National Health Program for 2016–2020, entitled “Comprehensive study of the mental health of the population and its contributing factors (EZOP II)” and conducted on a representative sample of 15,000 people, confirmed MBD in 26.5% of participants (i.e., approximately in 8,330,200 people) over their lifetime (29). It should be emphasized that these data were collected before the pandemic. During and after the pandemic, these rates might have been even higher (30).

Deaths are the most dramatic consequence of MBD. The findings of our study, reported in this manuscript, indicate that mortality due to MBD in Poland remained fairly stable between 2000 and 2014. Beginning in 2014, mortality increased rapidly. Evidence from numerous studies suggests a causal effect of average alcohol consumption on MBD (31–37). The observed increase in mortality due to MBD in Poland may be partly attributable to rising alcohol consumption.

The EZOP II study cited above shows that MBD caused by alcohol abuse were also among the most common problems in Poland (7.3%—2,307,700) (29). Our own study confirmed that this also applies to the frequency of deaths. Deaths caused by MBD due to alcohol abuse made up the greatest percentage, over 80% (and in the male group even over 90%).

Alcohol consumption in Poland has rapidly increased since the beginning of the 21st century (38). Rising alcohol consumption in Poland has led to a dramatic increase in mortality from alcohol-related diseases (39). It is estimated that around 30,000 deaths were caused by alcohol consumption in Poland in 2021 (40). This trend followed the decision of the Polish government to cut the excise duty on spirits by 30% in 2002 (40–42). In the years 2003–2008 annual vodka consumption rates doubled from 1.7 liters of pure alcohol per capita to 3.4 liters. In 2001 the rules on beer advertising have also been loosened (43, 44). This might have contributed to the rapid increase in beer consumption, from 3.7 liters of pure alcohol per capita in 2000 to 5.2 liters in 2008. Poland, traditionally a country with one of the highest levels of vodka consumption in Europe, also became one of the countries with the highest beer consumption, trailing only Czechia, Austria, and Germany. In 1989 annual beer consumption in Poland stood at 30 liters per capita, and in 2016 it was about 100 liters.

In 2010, the alcohol industry launched a marketing campaign promoting the sale of vodka in small bottles (SVBs) with a capacity of 100 mL or 200 mL, usually flavored. A report published by the research company Synergion in May 2019 estimated that more than 1 billion SVBs are sold every year (45). The growing popularity of SVBs may be the result of a combination of easy availability, wide variety of flavors catering to a range of tastes, convenience, and their discrete character. SVBs can be consumed easily and quickly (41).

At the same time, alcohol-related mortality declined in several other countries in the region, including Russia and Lithuania, where new measures to control alcohol consumption were introduced (46).

The greater the economic availability of alcohol, the greater its consumption. Hence, implementing an appropriate pricing policy is one of basic instruments for reducing health damage caused by alcohol. The European Commission commissioned RAND Europe to conduct a study on the affordability of alcohol products across the EU, and on the potential impacts of affordability on harmful use of alcohol. On this basis, the study is intended to provide evidence on whether alcohol affordability could be a useful policy lever to public authorities seeking to reduce harmful alcohol consumption in Europe. As a result of the conducted study, it was demonstrated that there is a statistically significant positive relationship between alcohol affordability (a composite measure examining the effect of price and income) and consumption across the EU. It was found that a 1% increase in availability leads to a total increase in alcohol consumption of 0.32% (47).

An indicator of economic accessibility of alcoholic beverages is the number of bottles of particular types of alcoholic beverages that can be purchased for an average monthly salary. Between 2002 and 2024, the economic availability of alcohol in Poland steadily increased. In 2002, the average monthly salary could buy 796 half-liter bottles of beer or 90 half-liter bottles of 40% vodka. In 2024, this number increased more than 2.5 times - to 2,103 bottles of beer or 238 bottles of vodka (38). Thus, it can be assumed that the state pricing policy does not promote the reduction of effects of harmful alcohol consumption.

In a study conducted by Ciabiada-Bryła et al., covering the years 1999–2017, the years of life lost (YLL) among Polish residents attributable to deaths resulting from excessive alcohol consumption were estimated (48). The largest number of YLLs were lost due to alcoholic liver disease (K70), a total of 1,630,592.41 years (SEYLL_p_ = 224.01 per 100,000 population), which accounted for 42.27% of all YLLs caused by excessive alcohol consumption. This disease contributed to 39.27% of YLLs in the male group and 57.87% of YLLs in the female group. The second most significant cause of YLLs was MBD due to alcohol use. The SEYLL value was 1,092,278.58 (SEYLL_p_ = 150.05), which accounted for 28.32% of YLLs (29.75% in men and 20.90% in women).

The results of other studies comparing mortality due to excessive alcohol consumption (including MBD-related mortality) with other European countries showed a worse situation in Poland than in the Czech Republic, Austria, Spain (49), and Ukraine (50).

In 2020, the first year of the COVID-19 pandemic, the highest mortality rates due to MBD caused by alcohol were recorded in the entire 24-year observation period (51). It appears that the pandemic exacerbated the long-standing problem of excessive alcohol consumption. Prior research established that psychological distress and problematic alcohol consumption often co-occur and major factors in disordered drinking are social isolation (52) and stress (53). A review by Rehm et al. explored previous public health crises and economic crises on alcohol consumption (54). They suggested two opposite outcomes during the pandemic were possible: an increase in alcohol use in some populations due to the psychological distress experienced, or a decrease in use due to limited availability and financial constraints. In a general population cross-sectional study in Poland, a higher tendency to drink more was found among alcohol addicts compared to non-addicts (55); and individuals who had current suicidal thoughts were more likely to drink more alcohol than before the pandemic than those without such thoughts (56). We should also point out a possible limited access to therapy and treatment for people with alcohol abuse (57).

Dementia makes up the second most common cause of death due to MBD. It should be emphasized that the Polish system for coding causes of death does not employ code F00 to denote Dementia in Alzheimer’s disease. All such deaths are classified as Diseases of the nervous system and are assigned code G30—Alzheimer’s disease. In the years 2000–2023, a total of 49,499 individuals in Poland died of Alzheimer’s disease, and the annual number of deaths increased from 1,001 in 2000 to 3,072 in 2023.

Findings from multiple studies consistently identify age as a predictor of mortality from dementia and Alzheimer’s disease (58–61). The increase in life expectancy and the associated growth in the proportion of individuals in the oldest age groups in Poland may therefore contribute to the rising mortality from dementia and Alzheimer’s disease.

The economic transformation, which occurred in Poland after 1989, substantially contributed to lifestyle and health behaviors of the Polish society (62). A health improvement caused by the development of new medical technologies and modern diagnostic methods contributed to many health rates such as a decrease in the mortality rate, which in turn, led to an increase in the average life expectancy. Between 2000 and 2023, life expectancy in Poland increased by 5 years for men (from 69.7 to 74.7) and by 4 years for women (from 78.0 to 82.0). Consequently, over the same period, the proportion of individuals aged 70 years or older rose by 4.6 percentage points among men (from 5.9 to 10.5%) and by 5.8 percentage points among women (from 10.3 to 16.1%) (63).

Since the end of World War II, the birth rate in Poland has fluctuated; it has either increased or decreased. The highest increase in the country’s population occurred between 1950 and 1958, approximately by 3.0 million people. The rapid increase in mortality from dementia since 2011 may be associated with the post-war baby boom generation in Poland reaching old age.

Also in this case, the exceptional impact of the COVID-19 pandemic on people with Alzheimer’s disease and other dementias should be noted. Studies have shown that despite taking into account advanced age and comorbidities such as hypertension and diabetes, people with dementia were more susceptible to COVID-19 infection than people without dementia. Furthermore, older people with dementia, especially those living in nursing homes, were at high risk of exacerbation of mental symptoms and serious behavioral disorders, being a result of social isolation during the pandemic (64–66). Currently, the war in Ukraine, which is causing a lot of concern, especially among the older generation, and has a negative impact on mental health, is another challenge.

Our study identified an increase in mortality due to schizophrenia in Poland beginning in 2012. However, given the low SDR values for schizophrenia, these findings should be interpreted with caution. Evidence from several studies and meta-analyses indicates that mortality among individuals with schizophrenia is higher than in the general population and that this excess mortality has not declined over time (67–69). Potential contributing factors include lifestyle-related behaviors, comorbid conditions, and adverse effects of antipsychotic treatment (70). In our study, the median age at death among individuals who died from schizophrenia in Poland in 2023 was 66 years for men and 71 years for women. Accordingly, life expectancy among individuals with schizophrenia was shorter by 8.7 years for men and by 11 years for women compared with the general population (63).

Deaths due to MBD mainly affect older people. However, it should be remembered that mental health problems very often begin in childhood or early youth, so prevention measures should be implemented at a very early age (71). Unfortunately, data on the mental health of young people are alarming. The EZOP II study shows that over half a million children and adolescents suffer from MBD. Over 200,000 sufferers are children aged 7–11 years and over 350,000 are adolescents aged 12–17 years. Internalizing disorders, which mainly include anxiety disorders, affect over 300,000 children and adolescents. Mood disorders, including depressive disorders and episodes of mania, have occurred in approximately 70,000 children and adolescents. Externalizing disorders have been experienced by approximately 300,000 children and adolescents. Of this number, over 100,000 are related to the use of psychoactive substances (alcohol or other psychoactive substances) (28).

According to data from the Supreme Medical Chamber (as of July 1, 2025), there are 582 active child and adolescent psychiatrists practicing in Poland, which translates to 8.6 doctors per 100,000 minors, or over 11,000 potential patients per one doctor (72, 73). These data indicate that the availability of psychiatric care for adolescents in Poland is insufficient. With regards to adults, the psychiatric care is slightly better. The number of practicing psychiatrists is 4,805, or 15.6 per 100,000 adult population of Poland. Psychiatrists account for 4.1% of all medical specialists. There is one psychiatrist per 6,400 adults. In contrast, in Europe in 2021, the average number of psychiatrists per 100,000 population was almost 17.0, ranging from 6.6 in Turkey to 52.8 in Switzerland (74, 75).

## Limitations

When citing data on the prevalence of MBD and related deaths, it is important to remember that these figures are often underestimated. Many individuals with mental health problems avoid seeking medical care due to limited knowledge about mental health, difficulties accessing healthcare services, and the risk of stigmatization. Accurately estimating mortality rates for these disorders is also challenging, as they are frequently not recorded as the cause of death on death certificates, despite being a significant contributing factor.

In Poland, as in most countries, causes of death are coded based on the so-called underlying cause—the disease that directly triggered the pathological process leading to death. Although this approach theoretically reduces coding errors, not all causes of death are correctly recorded, which may lead to underestimation.

In 2009, changes were introduced to standardize the coding of causes of death for statistical analyses. The physician certifying the death is responsible for completing the death certificate, indicating the underlying, intermediate, and immediate causes of death, while qualified teams of physicians code the causes according to the ICD-10 classification.

Increased life expectancy and the growing number of older adults have amplified the phenomenon of multimorbidity, defined as coexistence of multiple chronic diseases (76). In this context, the traditional approach based on a single underlying cause of death is increasingly inadequate in analysis of complex pathological processes observed in aging populations. The multiple causes of death (MCoD) approach allows for consideration of all diseases and health conditions that contributed to health deterioration and ultimately led to death.

Currently, MCoD analysis is not feasible in Poland. Due to procedures ensuring the confidentiality of medical information on deceased individuals, all data other than ICD codes for underlying causes of death are destroyed at the final stage of data processing. An exception was made in 2013 to test an automated system for coding causes of death: scans of death certificates issued on that year were preserved and subsequently used for the first MCoD analysis in Poland (77). Results demonstrated that the MCoD approach enables to determine the impact of diseases that are rarely coded as underlying causes of death but nonetheless significantly contribute to overall mortality, including MBD as well as dementias other than Alzheimer’s disease.

It may not be possible to carry out analyses on multiple causes of death unless death certificates are issued digitally on a large scale.

## Conclusion

Since 2014, mortality due to MBD has risen sharply in Poland, representing a pressing public health concern. The increasing demand for mental health services underscores the need to expand the mental health workforce and strengthen psychiatric care infrastructure to ensure broad access to specialized services.

The rising MBD mortality attributable to alcohol consumption calls for immediate, decisive, and evidence-based policy interventions. These should include regulatory measures to limit alcohol availability and consumption, complemented by comprehensive public health campaigns aimed at reducing social acceptance of harmful drinking and raising awareness of its severe health consequences.

Ongoing monitoring of MBD-related mortality is essential to guide policy and evaluate the effectiveness of interventions.

## Data Availability

The raw data supporting the conclusions of this article will be made available by the authors, without undue reservation.
